# CD25 expression could be a prognostic marker of bexarotene monotherapy for cutaneous T‐cell lymphomas

**DOI:** 10.1002/ski2.222

**Published:** 2023-02-12

**Authors:** Jun Yamamoto, Kentaro Ohuchi, Ryo Amagai, Yuna Roh, Junko Endo, Hiromu Chiba, Erika Tamabuchi, Yumi Kambayashi, Akira Hashimoto, Yoshihide Asano, Taku Fujimura

**Affiliations:** ^1^ Department of Dermatology Tohoku University Graduate School of Medicine Sendai Japan

## Abstract

Bexarotene is often administered to phototherapy‐resistant early cutaneous T‐cell lymphoma (CTCL) patients as one of the first‐line therapies in real‐world practice. Since bexarotene reduces the expression of CCR4 in CTCL cells and CCL22 to decrease serum CCL22 levels, bexarotene inhibits the migration of CTCL cells, as well as other CCR4+ cells, such as cytotoxic T cells and regulatory T cells, in the lesional skin of CTCL. In this report, the efficacy of bexarotene in 28 cases of CTCL, as well as its correlations with immunohistochemical profiles of tumour‐infiltrating leucocytes (TILs), was retrospectively investigated. The overall response rate at 1 and 4 months for the total cohort was 70.8% (95% CI, 50.6%–86.3%) and 47.8% (95% CI, 29.2%–67.0%), respectively. The disease control rate for the total cohort at 4 months was 65.2% (95% CI, 44.8%–81.3%). The mean event‐free survival for all patients was 4.1 months (0.3–68.5 months). In addition, the immunoreactive cells were calculated using digital microscopy, suggesting that the ratio of CD25+ cells among TILs was significantly increased in patients who responded to bexarotene (*p* = 0.0209), whereas there were no significant differences in the ratios of CD8+ cells, granulysin+ cells, and Foxp3+ cells among TILs between responder and non‐responder patients. Collectively, the ratio of CD25 expression among TILs might be a predictive biomarker for the efficacy of bexarotene.

## Graphical abstract

Bexarotene is often administered to phototherapy‐resistant early cutaneous T‐cell lymphoma (CTCL) patients as one of the first‐line therapies in real‐world practice. In this report, the efficacy of bexarotene in 28 cases of CTCL, as well as its correlations with immunohistochemical profiles of tumour‐infiltrating leucocytes (TILs), was retrospectively investigated. Present study suggested that the ratio of CD25 expression among TILs might be a predictive biomarker for the efficacy of bexarotene.

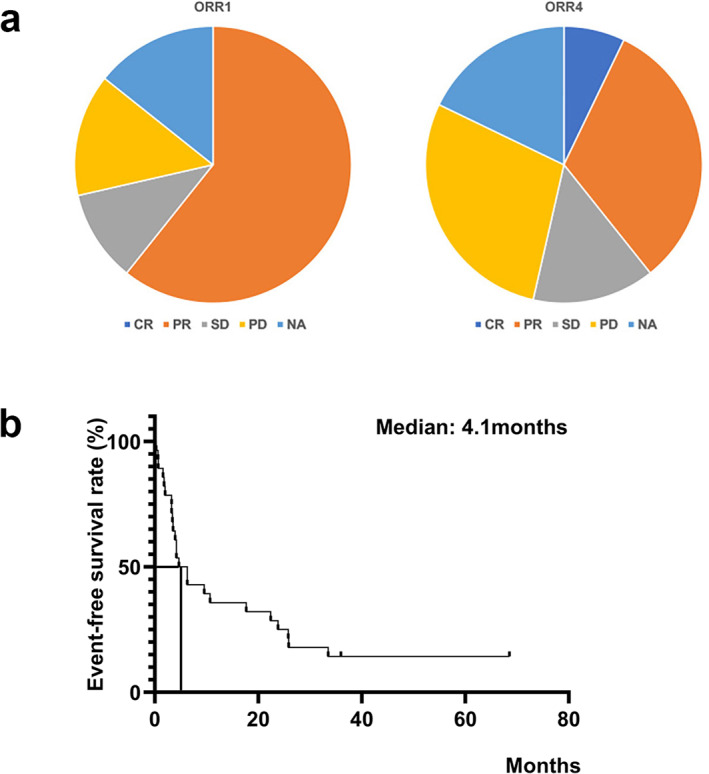

1

**What is already known about this topic?**

- Bexarotene is administered to phototherapy‐resistant early cutaneous T‐cell lymphoma (CTCL) patients as one of the first‐line therapies in real‐world practice.
- Various anti‐CTCL mechanisms of bexarotene, including immunomodulatory effects, have been reported.

**What does this study add?**

- Quantitative analysis of immunohistochemical staining showed that the ratio of CD25+ cells among tumour‐infiltrating leucocytes (TILs) was significantly increased in patients who responded to bexarotene.
- In contrast to CD25, there was no significant difference of the ratio of CD8+ cells, granulysin+ cells, and Foxp3+ cells among TILs.
- The ratio of CD25 expression among TILs might be a predictive biomarker for the efficacy of bexarotene.

## 2 INTRODUCTION

Bexarotene is often administered to phototherapy‐resistant, early cutaneous T‐cell lymphoma (CTCL) patients as one of the first‐line therapies in real‐world practice,^1^ and various anti‐CTCL mechanisms of bexarotene, including immunomodulatory effects, have been reported.^2^, ^3^, ^4^, ^5^ Of them, bexarotene reduces the expression of CCR4 in CTCL cells to inhibit the migration of CCR4‐expressing atypical cells in the lesional skin of CTCL.^3^ In addition, bexarotene reduces CCL22 production by M2 macrophages, which decreases serum CCL22 levels, leading to inhibition of the migration of CTCL cells in the lesional skin of CTCL to suppress CTCL disease activity.^2^, ^4^ Other in vitro experiments indicate that bexarotene suppresses not only the chemotaxis of CTCL cells, but also cell proliferation of CTCL cells through growth arrest and apoptosis.^5^, ^6^ Notably, since CCR4 expresses not only CTCL cells, but also skin‐homing T cells including CD8+ T cells and regulatory T cells (Tregs),^7^, ^8^ bexarotene might modulate the profiles of tumour‐infiltrating lymphocytes (TILs) to induce an anti‐CTCL immune response in CTCL patients. In this report, the efficacy of bexarotene against CTCL and its correlations with immunohistochemical (IHC) profiles of TILs were retrospectively investigated.

## 3 PATIENTS AND METHODS

### 3.1 Ethics statement for human experiments

The protocol for this human study was approved by the ethics committee of Tohoku University Graduate School of Medicine, Sendai, Japan (permit no. 2021‐1‐1213). All methods were performed in accordance with the relevant guidelines and regulations. All patients provided written, informed consent prior to enrolment in the study.

### 3.2 Patients

A database collected by the dermatology departments at Tohoku University Graduate School of Medicine was retrospectively reviewed to identify 28 patients with CTCL who had been treated with bexarotene from September 2016 through August 2022 (Table 1). Disease stage was determined at the time of initiating bexarotene.

**TABLE 1:** Characteristics of patients with CTCL: Age, sex, subtype, tumour stage, and efficacy.

Median ages (years)	62
Gender	
Male	18
Female	10
Subtype	
Mycosis fungoides	15
PC‐PTCL	6
PC‐ALCL	4
CD30+ LPD	2
NKT cell lymphoma	1
Stage (mycosis fungoides)	
IB	3
IIA	2
IIB	7
IIIA	2
IVB	1
Initial dose	
300 mg/m^2^	26
300 mg/body	2
ORR1	
CR	0
PR	17
SD	3
PD	4
NA	4
ORR4	
CR	2
PR	9
SD	4
PD	4

Abbreviations: CR, complete response; LPD, lymphoproliferative disorder; NA, Not applicable; ORR, objective response rate; PC‐ALCL, primary cutaneous anaplastic large cell lymphoma; PC‐PTCL, primary cutaneous peripheral T cell lymphoma; PR, partial response; SD, stable disease.

### 3.3 Treatment schedule and response assessment

Bexarotene alone was given orally at 300 mg/m^2^/day (26 cases) or at 300 mg/body (2 cases). The key efficacy end‐point was the evaluation of skin responses using the modified severity‐weighted assessment tool. The objective response rate (ORR) was defined as the percentage of patients showing either complete response or partial response. The disease control rate (DCR) was defined as the percentage of patients showing CR, PR, or stable disease (SD). ORR1 and ORR4 were defined as the percentages of objective response at 1 and 4 months, respectively, after the initial treatment. Event‐free survival (EFS) was defined as the duration of bexarotene administration without disease progression or any events causing discontinuation of bexarotene.

### 3.4 Safety assessment

Safety assessments involved the collection of data on adverse events (AEs), results of clinical laboratory tests and physical examinations, and vital signs. Severity grade (Common Terminology Criteria for AEs version 4.0—Japan Clinical Oncology Group) and the relationship to bexarotene were determined for each AE.

### 3.5 Patients, tissue samples, and IHC staining

Archival formalin‐fixed, paraffin‐embedded skin specimens that were collected at the initial visit from 21 CTCL patients who were treated in the Department of Dermatology at Tohoku University Graduate School of Medicine were evaluated (Table S1). Pathologists and dermatologists in each institute had diagnosed these patients with CTCL both clinically and pathologically. Mouse monoclonal antibodies (Abs) for human CD8 (DAKO, CA), CD25 (Nichirei Biosciences), and granulysin (MBL) and monoclonal rabbit Foxp3 (Abcam) were used for IHC staining. To quantify the IHC staining of each sample, the positive cells were counted using a BZ‐X800 (KEYENCE). The percentage of IHC‐positive cells per all tumour‐infiltrating cells was automatically counted.^9^

### 3.6 Statistical methods

For each group, ORR, DCR, and EFS and 95% confidence intervals were calculated. For a single comparison of two groups, the Mann‐Whitney *U*‐test was used.

Receiver‐operating characteristic (ROC) curves were established to evaluate cells positive for CD8, CD25, granulysin, and Foxp3 in patients administered bexarotene and used to calculate cut‐off values for cells positive for each of them; the areas under the curves were also determined. ROC curves. All statistical analyses were performed using JMP version 14.1 software (SAS Institute). The level of significance was set at *p* < 0.05.

## 4 RESULTS

### 4.1 Demographic data

Patient demographic data are shown in Table 1. The patients were 18 men and 10 women, with a mean age of 62.0 years. Subtypes of CTCL were as follows: mycosis fungoides (MF) in 15 cases; primary cutaneous peripheral T‐cell lymphoma in 6 cases; primary cutaneous anaplastic large‐cell lymphoma in 4 cases; CD30+ lymphoproliferative disorders in 2 cases; and NKT cell lymphoma in 1 case. With MF, 5 cases were early stage (IB‐IIA), and 10 cases were advanced stage (IIb, *n* = 7; IIIA, *n* = 2; IVA2, *n* = 1).

### 4.2 Efficacy of bexarotene

**FIGURE 1:**
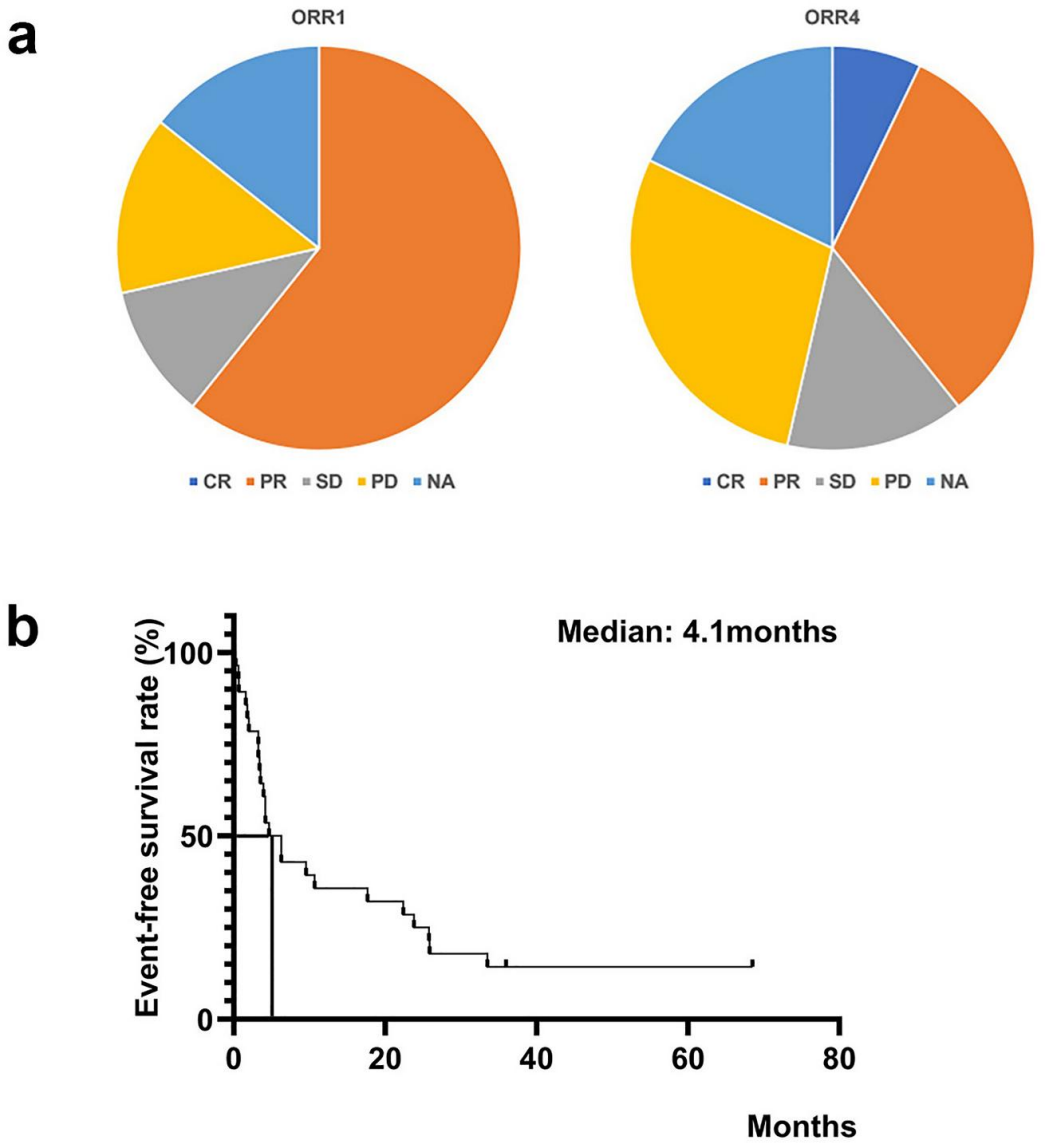
Efficacy of bexarotene for CTCL: ORR1 and ORR4 of bexarotene monotherapy (a) and EFS of bexarotene monotherapy (b). CTCL, cutaneous T‐cell lymphoma; EFS, event‐free survival.

Clinical response and EFS are summarized in Figure 1. ORR1 and ORR4 for the total cohort were 70.8% (95% CI, 50.6%–86.3%) and 47.8% (95% CI, 29.2%–67.0%), respectively (Figure 1a). ORR1 and ORR4 for the MF cohort were 76.9% (95% CI, 49.1%–92.5%) and 41.7% (95% CI, 19.3%–68.1%), respectively. ORR1 and ORR4 for the non‐MF cohort were 53.8% (95% CI, 29.–76.8%) and 54.5% (95% CI, 28.0%–78.7%), respectively. DCR for the total cohort at 4 months was 65.2% (95% CI, 44.8%–81.3%). DCR for the MF cohort was 66.7% (95% CI, 33.8%–86.4%), and that of the non‐MF CTCL cohort was 63.6% (95% CI, 35.2%–85.0%). Mean EFS for all patients was 4.1 months (0.3–68.5 months) (Figure 1b). Mean EFS for the MF cohort was 3.9 months (0.3–33.5 months), and that of the non‐MF CTCL cohort was 8.7 months (0.3–68.5 months).

### 4.3 Safety profile

**FIGURE 2:**
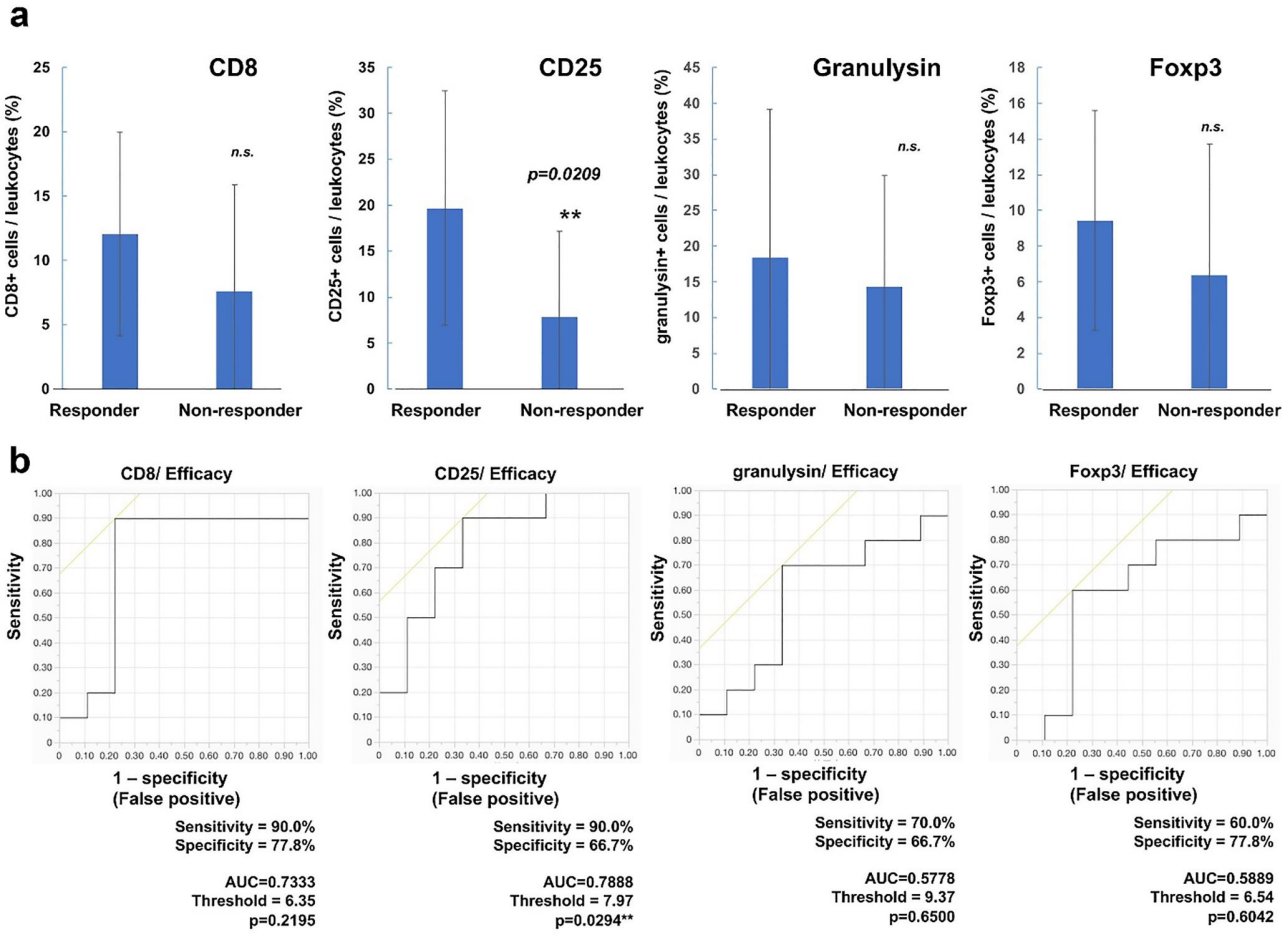
Quantitative analysis of CD8+ cells, CD25+ cells, granulysin+ cells and Foxp3+ cells: the percentage of immunoreactive cells in three representative areas was evaluated (a). The ROC curve was used to calculate cut‐offs for percentage of immunoreactive cells and the AUC (b). Cut‐offs were determined to distinguish vitiligo patients from no vitiligo patients or responders from non‐responders using Youden's index. AUC, areas under the curves; ROC, receiver‐operating characteristic.

Safety profiles for each cohort are shown in Figure 2. The incidence rate of total AEs for all patients was 100%. The incidence rate of severe or serious (S)AEs (G3, G4) for all patients was 57.1% (16 of 28 cases). The detailed profiles of AEs are shown in Table 2.

**TABLE 2:** Safety profile of each cutaneous T‐cell lymphoma case.

	G1/G2	G3/G4
Hyperlipidaemia	16 (57.1%)	12 (42.9%)
Leukocytopenia	7 (25%)	4 (14.3%)
Hypothyroidism	27 (96.4%)	‐
Fatigue	3 (10.7%)	‐
Headache	1 (3.6%)	‐
Drug eruption	‐	2 (7.1%)

### 4.4 IHC analysis of TILs in CTCL

Since bexarotene suppresses CCL22/CCR4‐related chemotaxis,^3^, ^4^ bexarotene might modulate the profiles of TILs to induce an anti‐CTCL immune response in CTCL patients. To test our hypothesis, IHC staining of CD8, CD25, granulysin, and Foxp3 was performed for 21 cases of CTCL treated with bexarotene (Table 1). The immunoreactive cells were calculated using digital microscopy, suggesting that the ratio of CD25+ cells among TILs was significantly increased in patients who responded to bexarotene (*p* = 0.0209), whereas there was no significant difference in the ratio of CD8+ cells, granulysin+ cells, and Foxp3+ cells between responder and non‐responder patients (Figure 2a). The threshold for the ratio of CD25+ cells to distinguish patients who responded to bexarotene was 7.97%, with sensitivity of 90.0% and specificity of 66.7% (*p* = 0.0294; Figure 2b).

## 5 DISCUSSION

Bexarotene, a third‐generation retinoid X receptor‐selective retinoid, is useful for both early and advanced CTCL,^10^, ^11^, ^12^ and it is recommended by the NCCN as an effective systemic therapy for MF with higher skin disease burden (beyond stage IIA).^13^ The ORR was 45% (25/56) for the treatment of refractory advanced‐stage CTCL patients dosed at 300 mg/m^2^, and the median duration of response was 299 days.^10^ In a Japanese population, the ORR at 24 weeks was 61.5% (8/13) as assessed using mSWAT in a phase I/II clinical trial,^14^ and 76.2% (19/29) for CTCL patients dosed at 300 mg/m^2^ in a multi‐centre, retrospective study.^12^ ORR1 and ORR4 were 70.8% (95% CI, 50.6%–86.3%) and 47.8% (95% CI, 29.2%–67.0%), respectively, and mean EFS for all patients was 4.1 months (0.3–68.5 months) in the present cohort. Since all the cases in present study administered oral bexarotene monotherapy, these response rate could be improved by the combination with such as low dose total electron beam therapy^15^ or phototherapy.^16^ Hyperlipidaemia is the most common SAE (42.8%), followed by leukocytopenia (10.7%). Although the incidence rate of SAEs was higher, their profiles were comparable to previous reports.^10^, ^11^, ^12^, ^14^ Since the efficacy of bexarotene and profiles of SAEs in the present study were comparable to the result of the clinical trial described above, the following IHC study appears to confirm previous reports.

CD25, the IL‐2Rα chain, is expressed on activated T cells and Tregs in healthy donors.^17^, ^18^ CD25 expression is prominent in human Tregs together with the expression of CCR4 and Foxp3,^17^ and the expression of CD25 is much higher in human Tregs than in activated T cells.^18^ On the other hand, atypical cells in MF lack CD7 expression, but highly express CD25 and Ki67.^19^ Indeed, in the lesional skin of MF, the ratio of Foxp3 on CD25+ cells is higher than in eczematous dermatitis,^20^ suggesting that the ratio of Tregs among CD25+ cells might be higher in MF than in healthy controls. Notably, Tregs are recruited to tumour tissues via chemokines, such as CCL22 binding to CCR4 expressed by Tregs, to induce an immunosuppressive tumour microenvironment.^17^ Since bexarotene reduces both the expression of CCR4 in CTCL cells and CCL22 production from TAMs to inhibit the migration of CCR4‐expressing cells,^13^, ^14^ investigating the subset of CCR4‐expressing cells in the lesional skin of CTCL might help predict the efficacy of bexarotene. Indeed, quantitative analysis of IHC staining showed that the ratio of CD25+ cells among TILs was significantly increased in patients who responded to bexarotene (*p* = 0.0209). In contrast to CD25, there was no significant difference of the ratio of CD8+ cells, granulysin+ cells, and Foxp3+ cells among TILs in the present study. Collectively, the ratio of CD25 expression among TILs might be a predictive biomarker for the efficacy of bexarotene. Moreover, our present data also suggested that ratio of CD25 expression among TILs might be a biomarker for Tregs‐targeting immunotherapy such as mogamulizumab (humanized anti‐CCR4 antibody), which is one of the most effective systemic therapies for relapsed CTCL.^2^, ^21^ Since the number of enroled CTCL patients is limited, further cases are needed to confirm this observation.

## CONFLICT OF INTEREST STATEMENT

None to declare.

## AUTHOR CONTRIBUTIONS

**Jun Yamamoto**: Data curation (equal); Resources (equal); Writing – original draft (equal). **Kentaro Ohuchi**: Data curation (equal); Resources (equal). **Ryo Amagai**: Resources (equal). **Yuna Roh**: Data curation (equal). **Junko Endo**: Data curation (equal). **Hiromu Chiba**: Resources (equal). **Erika Tamabuchi**: Resources (equal). **Yumi Kambayashi**: Resources (equal). **Akira Hashimoto**: Resources (equal). **Yoshihide Asano**: Resources (equal); Supervision (equal); Writing – review & editing (equal). **Taku Fujimura**: Conceptualization (equal); Data curation (equal); Formal analysis (equal); Funding acquisition (equal); Investigation (equal); Methodology (equal); Resources (equal); Supervision (equal); Writing – original draft (equal); Writing – review & editing (equal).

## ETHICS STATEMENT

The protocol for this human study was approved by the ethics committee of Tohoku University Graduate School of Medicine, Sendai, Japan (permit no. 2021‐1‐1213). All methods were performed in accordance with the relevant guidelines and regulations. All patients provided written, informed consent prior to enrolment in the study.

## Supporting information

Table S1

## Data Availability

The data that support the findings of this study are available on request from the corresponding author. The data are not publicly available due to privacy or ethical restrictions.
