# Supplementary material for: CD25 expression could be a prognostic marker of bexarotene monotherapy for cutaneous T‐cell lymphomas
Source: Skin Health Dis. 2023 Feb 12;3(3):e222. doi: 10.1002/ski2.222 (PMC10233073; doi:10.1002/ski2.222)
Supplement: Supplementary file 1 — Table S1 [file SKI2-3-e222-s001.docx]

Supplemental table

Quantitative analysis of IHC staining

　 CD8 (%) CD25 (%) Granulysin (%) Foxp3 (%)

Case 1 1.621 30.754 9.335 17.858

Case 2 6.353 8.136 15.765 0.711

Case 3 14.291 33.61 48.727 18.507

Case 4 5.621 1.23 1.153 2.64

Case 5 2.799 2.9 2.766 2.022

Case 6 3.566 8.451 8.879 1.444

Case 7

Case 8 0.287 7.974 0.861 6.544

Case 9 6.353 14.184 1.972 12.968

Case 10 4.944 1.146 31.477 1.752

Case 11 5.862 14.559 3.044 21.712

Case 12 9.595 13.258 7.622 19.203

Case 13

Case 14

Case 15

Case 16 10.189 10.682 9.371 11.689

Case 17 0.402 0.062 50.594 5.651

Case 18 19.224 3.127 5.915 3.487

Case 19

Case 20 25.912 2.287 17.342 6.354

Case 21 5.502 19.507 41.632 39.737

Case 22 28.01 46.992 9.895 9.427

Case 23

Case 24 12.047 19.602 22.909 3.255

Case 25 15.82 26.923 57.086 1.659

Case 26 22.592 22.599 10.482 17.277

Case 27 7.839 1.277 3.826 3.588

Case 28
